# Exploring the Link Between Noncoding RNAs and Glycolysis in Colorectal Cancer

**DOI:** 10.1111/jcmm.70443

**Published:** 2025-02-24

**Authors:** Liang Xu, Yu Shen, Chuanqiang Zhang, Tongguo Shi, Xuejuan Sheng

**Affiliations:** ^1^ Neonatal Department, Suzhou Ninth People's Hospital Suzhou Ninth Hospital Affiliated to Soochow University Suzhou Jiangsu China; ^2^ Department of General Surgery, Suzhou Ninth People's Hospital Suzhou Ninth Hospital Affiliated to Soochow University Suzhou Jiangsu China; ^3^ Department of General Surgery The Affiliated Jiangsu Shengze Hospital of Nanjing Medical University Suzhou China; ^4^ Shengze Clinical Medical College Kangda College of Nanjing Medical University Nanjing China; ^5^ Jiangsu Institute of Clinical Immunology The First Affiliated Hospital of Soochow University Suzhou China; ^6^ Health Management Center, Suzhou Ninth People's Hospital Suzhou Ninth Hospital Affiliated to Soochow University Suzhou Jiangsu China

**Keywords:** circular RNA, colorectal cancer, glycolysis, long noncoding RNA, microRNA, noncoding RNA

## Abstract

Glycolysis is implicated in the onset and progression of colorectal cancer (CRC) through its influence on the proliferation, invasiveness, chemoresistance and immune system evasion of neoplasm cells. Increasing evidence has shown that the abnormal expression of noncoding RNAs (ncRNAs), especially microRNAs (miRNAs), long noncoding RNAs (lncRNAs) and circular RNAs (circRNAs), in CRC is closely related to glycolysis. In this review, we present a synthesis of the latest research insights into the modulatory roles and distinct pathways of ncRNAs in the glycolytic process in CRC. This knowledge may pave the way for identifying novel therapeutic targets, as well as novel prognostic and diagnostic biomarkers for CRC.

## 1 Introduction

Colorectal cancer (CRC) is the third most commonly diagnosed cancer and the second leading cause of cancer‐related deaths globally [1]. Factors contributing to the risk of CRC encompass a dietary pattern, excessive body weight, a sedentary lifestyle, tobacco use, the intake of alcoholic beverages and familial predisposition [2]. Despite the advances in therapeutic methods such as surgical resection, targeted therapy, chemoradiotherapy and immunotherapy, the survival rates for patients with CRC are still suboptimal. This is largely attributable to the fact that CRC is often detected at later stages, when the disease has progressed to intermediate or advanced phases [3]. Hence, more research is needed to elucidate the underlying molecular mechanisms of CRC and help to discover and develop effective biomarkers and targets for CRC diagnosis and treatment.

The Warburg effect, initially characterised by Otto Warburg during the 1920s, is a hallmark of cancer metabolism [4]. Although glycolysis is less efficient than oxidative phosphorylation in terms of ATP production, cancer cells benefit from glycolysis in several ways. This metabolic reprogramming provides rapidly proliferating tumour cells with the ATP and biosynthetic precursors needed for growth and survival, while also creating a microenvironment that promotes tumour progression [5].

Noncoding RNAs (ncRNAs) are a category of RNA molecules that originate from DNA transcription but do not undergo translation into proteins [6]. Although they do not encode proteins, ncRNAs perform crucial regulatory functions across a spectrum of biological activities. They participate in modulating gene expression, altering chromatin structure, influencing RNA splicing and mediating epigenetic changes. The primary classifications of ncRNAs include circular RNAs (circRNAs), long noncoding RNAs (lncRNAs) and microRNAs (miRNAs) [6]. Emerging evidence highlights the critical role of ncRNAs across various malignancies, including CRC [7, 8]. Their capacity to influence diverse facets of tumorigenesis, including cellular proliferation, programmed cell death, invasive spread, therapeutic resistance and metabolic pathways, makes them potential therapeutic targets and biomarkers for CRC diagnosis and therapy [7, 8]. For example, Lee et al. reported that five key signalling pathways involved in CRC pathogenesis, the Wnt signalling pathway, the PI3K/AKT/mTOR signalling pathway, the MAPK signalling pathway, the TGF‐β signalling pathway and the p53 signalling pathway, are regulated by ncRNAs [9]. Recently, numerous studies have elucidated the important role of miRNAs, lncRNAs and circRNAs in regulating glucose metabolism in multiple cancers, including CRC [10, 11, 12]. However, few articles have systematically reviewed the roles of ncRNAs, including miRNAs, lncRNAs and circRNAs, in glycolysis and progression of CRC. This review summarises how ncRNAs regulate aerobic glycolysis in CRC, which may help to identify potential new therapeutic strategies for CRC therapy.

## 2 The Process of Glycolysis

**FIGURE 1:**
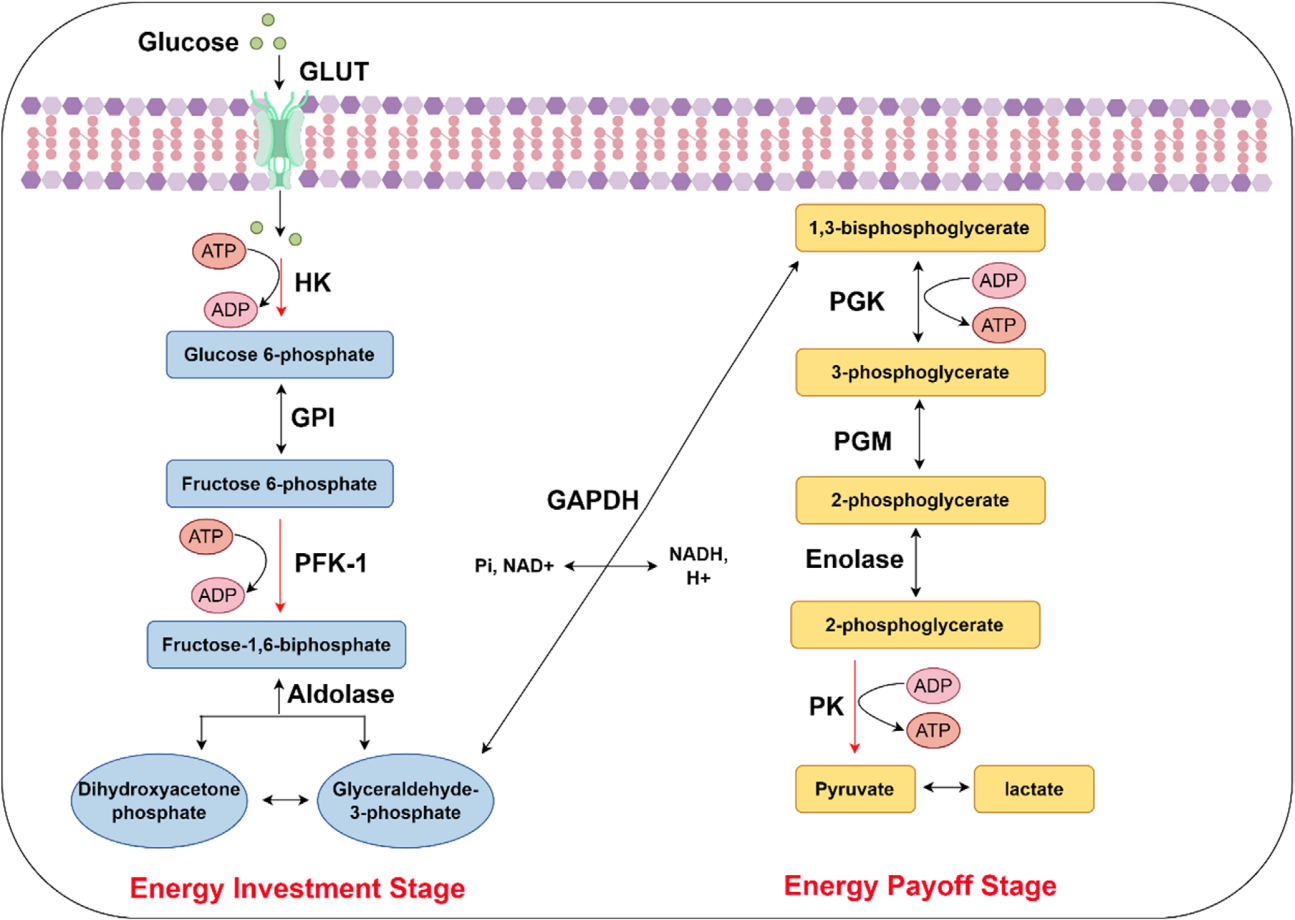
The key biochemical process of glycolysis in cells. The process of glycolysis involves the conversion of glucose into pyruvate, yielding energy in the form of ATP. This process begins with glucose, which is transported into the cell via glucose transporters (GLUTs). Once inside the cell, glucose undergoes a series of enzymatic reactions catalysed by glycolytic enzymes such as hexokinase (HK), phosphofructokinase‐1 (PFK‐1) and pyruvate kinase (PK). These reactions facilitate the production of ATP, thus providing energy to the cell. Glycolysis can be conceptually divided into two stages: the energy investment phase, where energy is invested in the form of ATP to prime glucose for subsequent reactions, and the energy payoff phase, where energy is generated as ATP from the conversion of glucose into pyruvate.

As a core metabolic process, glycolysis converts glucose, a hexose sugar, into two pyruvate molecules, each comprising three carbon atoms [13]. This metabolic sequence takes place in the cellular cytoplasm and is independent of oxygen, which makes it an anaerobic route. The Warburg effect involves a series of 10 enzyme‐catalysed steps and can be divided into two main stages: the energy investment stage and the energy payoff stage (Figure 1) [14].

In the first stage of glycolysis, two molecules of ATP are expended to phosphorylate glucose, converting it into fructose‐1,6‐bisphosphate. The initial phase involves the hexokinase (HK) enzyme, which facilitates the transformation of glucose into glucose‐6‐phosphate through phosphorylation [15]. Phosphoglucose isomerase subsequently catalyses the conversion of glucose‐6‐phosphate into fructose‐6‐phosphate. A critical regulatory step follows, in which phosphofructokinase‐1 (PFK‐1) modulates the phosphorylation of fructose‐6‐phosphate to yield fructose‐1,6‐bisphosphate [16]. Ultimately, aldolase divides fructose‐1,6‐bisphosphate into two molecules, each with three carbons: dihydroxyacetone phosphate (DHAP) and glyceraldehyde‐3‐phosphate (G3P). The triose phosphate isomerase subsequently facilitates the conversion of DHAP into an additional G3P molecule, thereby concluding the initial phase of energy expenditure [17].

In the energy payoff stage of glycolysis, ATP and NADH are produced as energy outputs. The two molecules of G3P generated in the energy investment stage are oxidised and converted into pyruvate through a series of reactions. First, G3P dehydrogenase (GAPDH) catalyses the oxidation of G3P, reducing NAD^+^ to NADH and forming 1,3‐bisphosphoglycerate [18]. Next, phosphoglycerate kinase (PGK) induces the transfer of a phosphate group from 1,3‐bisphosphoglycerate to ADP, leading to the formation of ATP and 3‐phosphoglycerate [19]. Through a series of additional rearrangements, the final steps yield another ATP molecule and ultimately result in the formation of pyruvate.

## 3 Glycolysis Regulates CRC Progression

In CRC, increased glycolytic activity is linked to tumour growth, metastasis, drug resistance and immune escape [20]. Key glycolytic enzymes, such as hexokinase 2 (HK2), pyruvate kinase M2 (PKM2) and lactate dehydrogenase A (LDHA), are often overexpressed in CRC cells, supporting their high metabolic demands [21]. Additionally, glycolysis produces the by‐product lactate, which contributes to the acidic tumour microenvironment that promotes immune evasion, angiogenesis and extracellular matrix remodelling, all of which facilitate tumour progression [22]. Hence, targeting glycolysis plays a significant role in inhibiting tumour cell proliferation, altering the tumour microenvironment, improving therapeutic efficacy and providing new ideas and strategies for cancer prevention and treatment.

### 3.1 Glycolysis Modulates the Proliferation of CRC Cells

Tumour cell growth is characterised by uncontrolled cell division and survival, which enable neoplastic cells to proliferate and infiltrate adjacent tissues. Glycolysis is a key factor in cancer cell proliferation because it not only sustains energy generation but also furnishes vital precursors for the biosynthesis of compounds essential for cellular growth [20]. For instance, protein arginine methyltransferase 1 (PRMT1) induces arginine asymmetric dimethylation of PGK1 at R206 (meR206‐PGK1) and increases the phosphorylation level of PGK1 at S203 (pS203‐PGK1), which promotes glycolysis, proliferation and tumorigenesis in CRC [23]. Moreover, the deubiquitinase OTUB2 directly interacts with PKM2 and inhibits its ubiquitination, resulting in enhanced glycolysis, proliferation and migratory capacity in CRC cells [24]. These studies suggest that elevated glycolysis promotes CRC cell proliferation.

### 3.2 Glycolysis Modulates the Migration and Invasion of CRC Cells

In CRC, the glycolytic axis is key for the cell proliferation of neoplasm cells and their ability to migrate and invade, which are essential for cancer metastasis [25]. Increased glycolytic activity in CRC cells generates ATP and metabolic intermediates, thus supplying the energy and building blocks needed for cell movement [26]. Furthermore, glycolysis‐induced signalling and transcription factors, including HIF‐1α, regulate the expression of genes associated with epithelial–mesenchymal transition (EMT), a process that enhances cell migration and invasiveness [27]. Our previous work revealed that HES1 promoted aerobic glycolysis in CRC cells by stabilising m6A‐modified *GLUT1* mRNA, which increased GLUT1 expression and promoted the migration and invasion of CRC cells [28]. Hence, blocking glycolysis in CRC is a potential strategy for reducing tumour cell migration and invasion, thereby limiting metastasis.

### 3.3 Glycolysis Modulates Drug Resistance in CRC

Resistance to drugs poses significant challenges in the efficacious management of CRC, limiting the efficacy of chemotherapeutic and targeted interventions [29]. Notably, glycolysis is implicated in the emergence of resistance to drugs in various cancers, including CRC. Tumour cells that rely heavily on glycolysis for energy production and biosynthesis exhibit metabolic adaptations that help them survive chemotherapy and targeted therapies [30]. Shuohui Dong and colleagues noted that 5‐fluorouracil (5‐FU)–resistant CRC had aberrant glycolysis. Their research indicated that the ROS/PI3K/AKT and Wnt/β‐catenin pathways stimulate HIF‐1α‐mediated glucose metabolism, which confers resistance to 5‐FU in CRC [31]. Furthermore, TRIP13 (thyroid hormone receptor interactor 13) promoted cell stemness by activating glycolysis, leading to increased resistance to DOX (doxorubicin) in CRC cells [32]. Consequently, glycolysis is pivotal in resistance to drugs in CRC cells. Integrating inhibitors of glycolysis with conventional therapeutic approaches could represent a potent strategy to counteract drug resistance in CRC.

### 3.4 Glycolysis Modulates Immune Escape

Glycolysis is instrumental in facilitating immune evasion in CRC, enabling the recognition and elimination of neoplastic cells by the immune system [33, 34]. Increased glycolysis in CRC cells aids immune evasion by several mechanisms, including suppressing immune cell functions (particularly those of T cells and natural killer [NK] cells) through the creation of an acidic environment, competing with effector immune cells for glucose, increasing the levels of immune checkpoint molecules and activating immunosuppressive cells such as regulatory T cells (Tregs) and myeloid‐derived suppressor cells (MDSCs) [33, 34]. For example, m6A‐modified circQSOX1 promoted CRC tumorigenesis by regulating the miR‐326/miR‐330‐5p/*PGAM1* axis, which further facilitated CRC immune escape by activating glycolysis [35]. Therefore, targeting glycolysis in CRC has emerged as a promising approach to disrupt the immune escape of CRC cells. By inhibiting glycolytic enzymes or glucose transporters (GLUTs), researchers aim to reduce lactate accumulation, relieve glucose competition and enhance the effectiveness of immunotherapies by restoring immune cell function in the tumour microenvironment.

**FIGURE 2:**
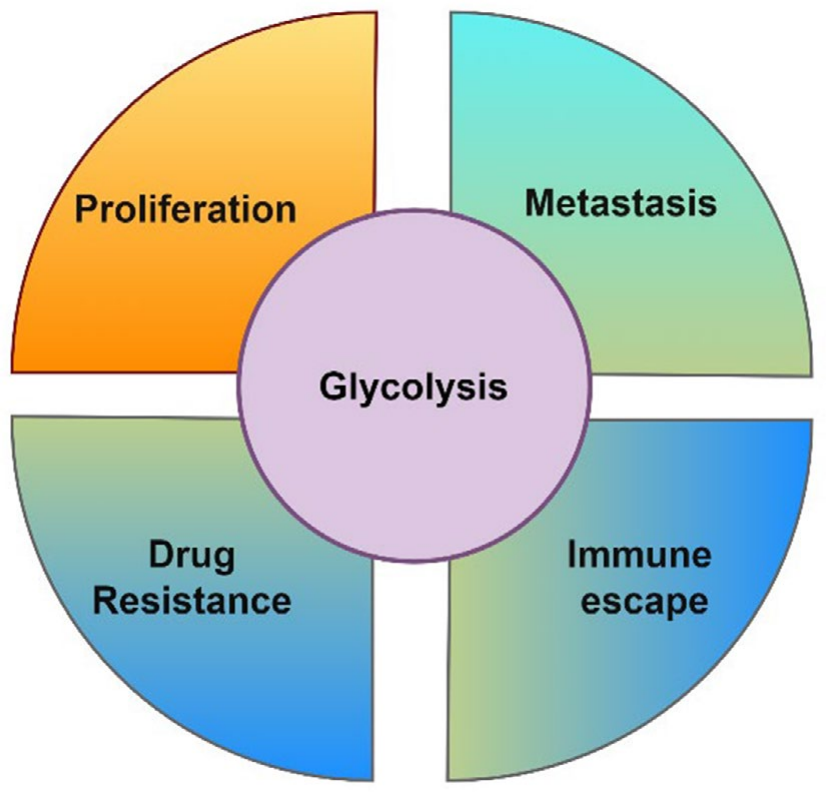
Glycolysis plays a pivotal role in regulating various biological functions in CRC. It is intricately linked to tumour growth, metastasis, drug resistance and immune evasion, which are critical hallmarks of CRC progression.

Overall, these findings indicate that targeting glycolysis in CRC presents a promising strategy to curb tumour growth, metastasis, drug resistance and immune escape (Figure 2), potentially improving therapeutic outcomes.

## 4 ncRNAs Regulate Glycolysis in CRC

**FIGURE 3:**
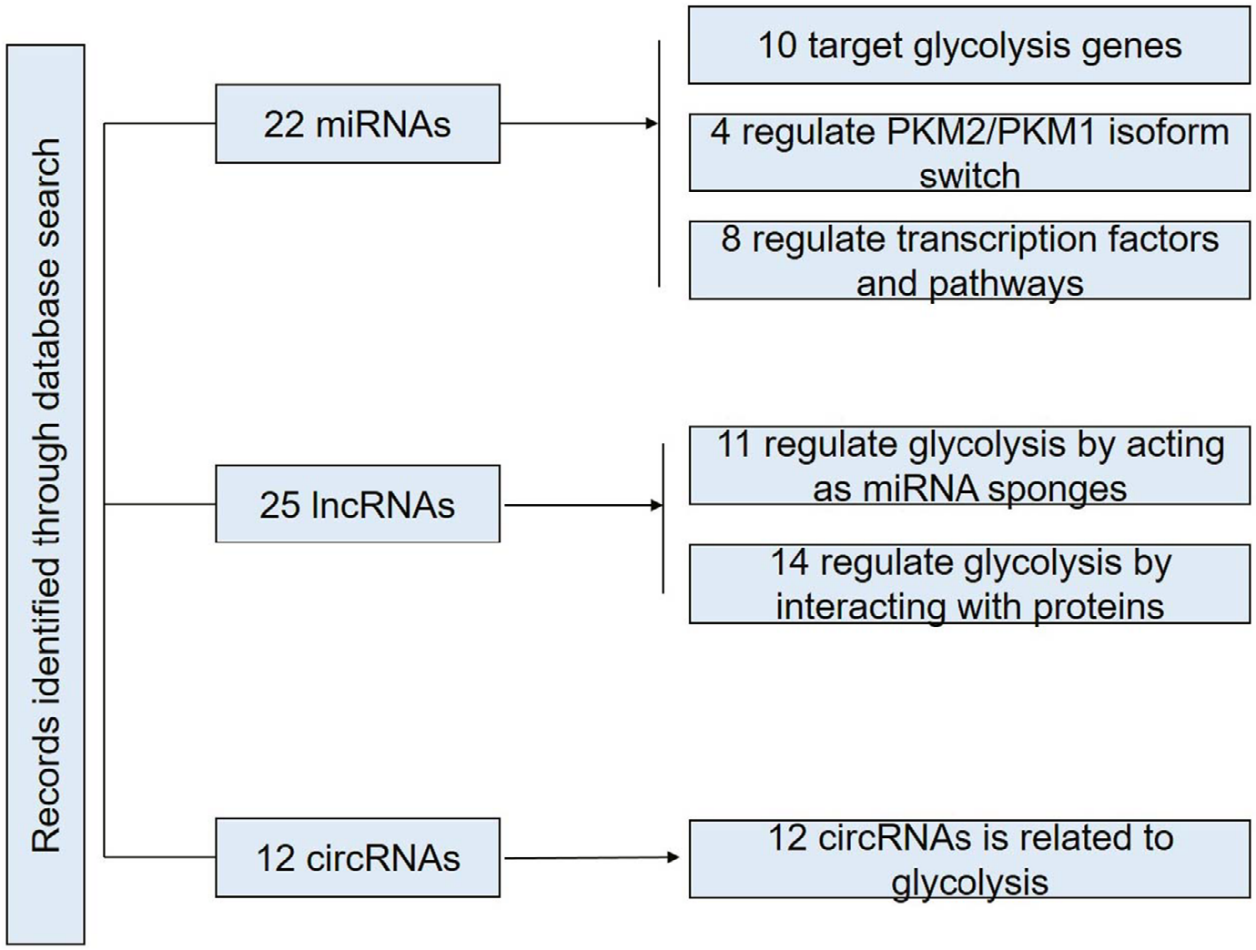
Flow diagram of study selection.

To assess the relationship between ncRNAs and glycolysis in CRC, we performed a systematic literature search that yielded a total of 59 citations (Figure 3). After reviewing the full texts, we identified 22 citations related to miRNAs, 25 citations related to lncRNAs and 12 citations related to circRNAs. Furthermore, we examined the effects of ncRNAs on glycolysis in CRC through various molecular mechanisms (Figure 3).

### 4.1 Dysregulation of miRNAs Is Related to Glycolysis in CRC

miRNAs, a family of small ncRNAs ranging from 18 to 25 nucleotides, act predominantly by targeting specific mRNAs, modulating their translation or inducing degradation, and are thus central to the regulation of gene expression. miRNAs are involved in many biological processes and signalling pathways, including cell differentiation, proliferation and apoptosis, and play a broad regulatory roles [36, 37]. Emerging research indicates a strong association between miRNAs and glycolytic activity in CRC, as summarised in Table 1.

**TABLE 1:** Overview of miRNAs regulating glycolysis in CRC.

miRNAs	Expression	Targets	Pathway	Phenotypes	Ref.
miR‐143	Down	HK2	/	5‐FU resistance	[38]
miR‐513a‐3p	Down	HK2	/	Proliferation	[39]
miR‐4458	Down	HK2	/	Proliferation	[40]
miR‐98	Down	HK2	/	Proliferation	[41]
miR‐6769b‐3p miR‐499a‐3p	/				
miR‐122	Down	PKM2	/	5‐FU resistance	[42]
miR‐142‐3p	Down	PKM2	/	Migration, invasion	[43]
miR‐148	Down	PFKFB3	/	Proliferation, metastasis, oxaliplatin/5‐Fu resistance	[44]
miR‐328	Down	GLUT1	/	/	[45]
miR‐6769b‐3p miR‐499a‐3p		GLUT3/PGAM1	/	Malignant phenotype	[46]
miR‐125b‐5p	Down	GLUT5	/	Migration, invasion, oxaliplatin and 5‐FU resistance	[47]
miR‐206	Down	hnRNPA1	PKM2/PKM1 shift	Proliferation	[50]
miR‐124 miR‐137 miR‐240	/	PTBP1/hnRNAPA1/hnRNAPA2	PKM2/PKM1 shift	/	[51]
miR‐339‐5p	/	hnRNPA1 PTBP1	PKM2/PKM1 shift	Kaempferol‐induced proliferation inhibition	[52]
miR‐326	/	hnRNPA1hnRNPA2PTBP1 PKM2	/	5‐FU resistance	[53]
miR‐23a∼27a∼24 cluster	Up	TCA cycle enzymes	/	Proliferation	[56]
miR‐130a‐3p	Up	LATS2 SAV1	YAP1/HIF1A axis	Proliferation, migration, invasion, angiogenesis	[57]
miR‐526b‐3p	Down	HIF‐1α	/	Proliferation, migration, invasion	[58]
miR‐181d	Up	CRY2 FBXL3	miR‐181d/CRY2/FBXL3/c‐myc feedback loop	Proliferation, migration, invasion	[60]
miR‐22	Down	MAX	/	Migration, invasion Cancer stem cell transcription factors	[61]
miR‐27a	Up	AMPK	AMPK pathway mTOR pathway	Proliferation Chemoresistance	[63]
miR‐181a	Up	PTEN	PTEN/AKT	Proliferation	[65]
miR‐135b	Up	USP13	USP13/PETN	Proliferation	[66]

#### 4.1.1 MiRNAs Directly Target Glycolysis Genes

Research has indicated that a number of proteins implicated in glycolysis, including HK2, PFKFB3 and PKM2, exhibit elevated expression levels in CRC and are subject to miRNA‐mediated regulation. HK2, which encodes a rate‐limiting enzyme in glycolysis, is responsible for dysregulated glycolysis in cancers. For instance, in 5‐FU–resistant colon cancer specimens and cell lines, miR‐143 is underexpressed, and studies have shown that it can hinder EGFR‐driven 5‐FU resistance and glycolysis in CRC cells by directly targeting HK2 [38]. The miRNAs miR‐4458, miR‐513a‐3p and miR‐98 are also downregulated in CRC, and their overexpression was found to curb the proliferation and glycometabolism of CRC cells by negatively modulating HK2 [39, 40, 41]. PKM2, a key enzyme in oncogenic metabolism, catalyses the terminal reaction of the glycolytic pathway. miR‐122 was found to negatively regulate PKM2 expression and re‐sensitise 5‐FU–resistant CRC cells to 5‐FU [42]. Ren et al. demonstrated that PKM2‐mediated aerobic glycolysis contributed to CRC cell invasion and migration induced by miR‐142‐3p [43]. The enzyme 6‐phosphofructo‐2‐kinase/fructose‐2,6‐bisphosphatase 3 (PFKFB3) phosphorylates F6P (fructose‐6‐phosphate) to F2,6BP (fructose‐2,6‐bisphosphate). In recurrent or metastatic CRC patients, miR‐488 levels are downregulated, leading to the suppression of cellular glucose absorption and lactate output; an increase in sensitivity to oxaliplatin and 5‐FU; and a reduction in the ability to proliferate, invade and migrate [44].

The GLUT family, encoded by the *SLC2A* gene, is mainly responsible for transporting various sugars across the cell membrane. The first class of GLUT transporters, including GLUT1, GLUT2, GLUT3 and GLUT4, mainly facilitates the movement of glucose into cells. MiR‐328, which is downregulated in CRC tissues, directly targets GLUT1, resulting in a metabolic shift in CRC cells [45]. Hou et al. reported that METTL14 inhibited aerobic glycolysis in p53 wild‐type CRC by downregulating GLUT3 and PGAM1 expression through the selective enhancement of the m6A‐YTHDF2–dependent processing of pri‐miR‐6769b and pri‐miR‐499a [46]. Park et al. reported that the activation of AKT3 and AKT1 in drug‐resistant CRC cells leads to the dysregulation of miR‐125b‐5p and the overexpression of GLUT5, which in turn increases the migration and invasion of CRC cells and bolsters their resistance to oxaliplatin and 5‐FU [47].

#### 4.1.2 MiRNAs Regulate PKM2/PKM1 Isoform Switch

PKM contains two isoforms, PKM1 and PKM2. Cancer cells often undergo a PKM2/PKM1 isoform switch, favouring the expression of PKM2, which supports the unique metabolic needs of tumour cells by allowing them to adjust pyruvate kinase activity according to cellular conditions [48, 49]. The forced expression of miR‐206 markedly curtails glycolysis and the proliferation of CRC cells by redirecting the PKM isoform transformation from PKM2 to PKM1, thereby inhibiting PKM2 production in a manner dependent on hnRNP1 [50]. Like miR‐137, the miRNAs miR‐124, miR‐206 and miR‐370 also influence the PKM1/PKM2 isoform switch in CRC cells by modulating the expression of alternative splicing factors, including PTB1, hnRNPA1 and hnRNPA2 [51]. Interestingly, kaempferol, a flavonoid found in numerous natural food sources, was found to reverse aerobic glycolysis and inhibit the proliferation of CRC cells via a miR‐339‐5p–mediated PKM1/PKM2 shift by targeting hnRNPA1 and PTBP1 [52]. In another study, miR‐326 was demonstrated to modulate the hnRNPA1/hnRNPA2/PTBP1‐PKM2 axis, potentially enhancing the responsiveness of CRC cells to 5‐FU through kaempferol [53].

#### 4.1.3 MiRNAs Regulate Important Transcription Factors and Pathways

Glycolysis in cancer cells is modulated by important transcription factors and pathways, such as the HIF‐1α, AMP‐activated protein kinase (AMPK) and c‐MYC pathway. As a crucial transcription factor in the cellular response to low oxygen levels, HIF‐1α is often activated in tumour environments where oxygen is limited [54]. In cancer, HIF‐1α acts as a pivotal factor in shifting cellular metabolism towards glycolysis, even under oxygenated conditions. By upregulating molecules involved in glycolysis, such as GLUT1, LDHA and HK2, HIF‐1α enhances glucose uptake and lactate production, providing cancer cells with energy and biosynthetic precursors essential for rapid growth and survival [54, 55]. The HIF‐1α–induced miR‐23a∼27a∼24 cluster promoted CRC progression by regulating the TCA cycle enzymes PDHB, PDHA1, DLD, IDH3A, CS, IDH1, ACLY, MDH1B and SDHA [56]. A study by Sun et al. revealed that miR‐103a‐3p, which is abundant in tumour cells, upregulates HIF‐1α by targeting SAV1 and LATS2, key components of the Hippo/YAP1 signalling cascade. This upregulation stimulates increased proliferation, invasion, migration, glycolytic activity and angiogenesis in CRC [57]. Moreover, miR‐526b‐3p, which acts as an oncosuppressor, reduces HIF‐1α levels in CRC cells, thereby inhibiting their proliferation and metastatic potential [58].

c‐MYC, an additional critical transcription factor that is frequently overexpressed in various cancers, is instrumental in enhancing glycolysis to meet the elevated energy requirements and swift replication of neoplastic cells [59]. Guo et al. elucidated that miR‐181d levels are elevated in CRC tissues and that its suppression curbs the migration, invasion and proliferation of CRC cells via the CRY2/FBXL3/c‐MYC regulatory loop [60]. The miR‐22, which is underexpressed in CRC cells and tissues, markedly limits migration, invasion and the production of tumour stem cell transcription factors in CRC cells by directly targeting MYC‐associated factor X (MAX) [61].

The AMPK pathway is pivotal in the modulation of glycolysis and serves as a central modulator of cellular energy homeostasis. In tumour cells, AMPK activation can sometimes suppress tumour growth by inhibiting abnormal metabolic patterns, such as the Warburg effect [62]. Barisciano and colleagues noted that the overexpression of miR‐27a inhibited AMPK activity and activated mTOR signalling, working alongside oncogenes and metabolic regulators in tumour cells to drive an aerobic glycolytic metabolism [63]. Moreover, the PI3K/AKT signalling cascade has been shown to participate in the control of glycolytic processes within cancer cells [64]. The overexpression of miR‐181a inhibits the expression of PTEN, resulting in an increase in phosphorylated AKT, which in turn triggers a metabolic shift and promotes cell proliferation in CRC [65]. Xiang et al. noted that miR‐135b decreased the levels of ubiquitin‐specific peptidase 13 (USP13), leading to a decrease in PTEN stability. Furthermore, the suppression of USP13 increased endogenous miR‐135b expression. As a result, miR‐135b, USP13 and PTEN form a positive feedback loop, promoting cell proliferation and glycolysis in CRC [66].

Collectively, these findings suggest that the dysregulation of miRNAs in glycolysis represents a promising area for developing innovative and effective strategies in the prevention and treatment of CRC.

**TABLE 2:** Overview of lncRNAs regulating glycolysis in CRC.

lncRNAs	Expression	Regulatory mechanism	Phenotypes	Ref.
LOXL1‐AS1	Up	ceRNA for miR‐1224‐5p/miR‐761 enhances HK2 expression	Proliferation, invasion, migration, apoptosis	[72]
FGD5‐AS1	Up	ceRNA for miR‐330‐3p enhances HK2 expression	5‐Fu resistance	[73]
MIR17HG	Up	ceRNA for miR‐186‐5p enhances HK1 expression	Liver metastasis	[74]
lncARSR	Up	ceRNA for miR‐34a‐5p increases HK1 expression	Migration, invasion	[75]
TMPO‐AS1	Up	ceRNA for miR‐1270 enhances PKM2 expression	Proliferation, invasion, migration, apoptosis	[76]
XIST	Up	ceRNA for miR‐137 increases the PKM2/PKM1 ratio	Proliferation, invasion, migration, 5‐FU/cisplatin resistance	[77]
LINC00174	Up	ceRNA for miR‐2467‐3p enhances ENO3 expression	Inflammation, proliferation, migration, invasion, apoptosis	[78]
ANRIL	Up	ceRNA for miR‐186‐5p enhances HIF‐1α expression	Proliferation, apoptosis	[125]
MCF2L‐AS1	Up	ceRNA for miR‐874‐3p enhances FOXM1 expression	Proliferation, migration, invasion	[80]
HIF1A‐AS2	Up	ceRNA for miR‐141‐3p enhances FOXC1 expression	Proliferation, invasion, migration	[82]
AC105118.1	Up	ceRNA for miR‐378a‐3p enhances KIF26B expression	Oxaliplatin resistance	[83]
SLCC1	Up	Interacts with AHR and regulates HK2 expression	Proliferation	[85]
lncRNA 495810	Up	Binds and increases the stability of PKM2	Proliferation, metastasis	[86, 87]
FEZF1‐AS1	Up	Binds and increases the stability of PKM2	Proliferation, metastasis	[88]
SNHG6	Up	Interacts with hnRNPA1 and increases the PKM2/PKM1 ratio	Proliferation	[89]
LINC01852	Down	Inhibits SRSF5‐mediated alternative splicing of PKM	Proliferation, chemoresistance	[90]
ENO1‐IT1	Up	Behaves as a guider modular for KAT7 histone acetyltransferase to regulate ENO1 expression	Carcinogenesis	[91]
AC005392.2	Up	Increases the stability of GLUT1	Vasculogenic mimicry	[92]
SNHG15	Up	Regulates the expression of TYMS, BCL2, GLUT1 and PKM2	Proliferation, 5‐FU resistance	[93]
GLCC1	Up	Interacts with HSP90 and increases the stability of c‐MYC	Cell survival, proliferation	[94]
LINRIS	Up	Increases the stability of and enhances c‐MYC expression	Proliferation	[95]
LINC01977	Up	Regulates ERK‐mediated phosphorylation and increases the stability of c‐MYC	Proliferation, metastasis	[96]
MEG3	Down	Induces ubiquitin‐dependent degradation of c‐Myc	Proliferation, migration, invasion	[97]
lnc‐RP11‐536 K7.3	Up	Recruits SOX2 to transcriptionally activate USP7 and increases the stability of HIF‐1α	Proliferation, angiogenesis, chemoresistance	[98]

### 4.2 Dysregulation of lncRNAs Is Related to Glycolysis in CRC

LncRNAs, which exceed 200 nucleotides in length, play many roles, such as influencing chromatin remodelling, modulating molecule expression at the transcriptional level and acting as platforms for assembling protein complexes [67]. LncRNAs have emerged as key players in cellular homeostasis and are implicated in various diseases, especially cancer, where they function as either oncogenes or tumour suppressors [68]. Importantly, lncRNAs are key regulators of glycolysis in multiple cancers. For example, H19, a well‐known lncRNA, is involved in tumour cell growth, proliferation, invasion, metastasis and glycolysis [69, 70]. Herein, we summarise the role and mechanism of lncRNAs in modulating glycolysis in CRC (Table 2).

#### 4.2.1 LncRNAs Regulate Glycolysis by Acting as miRNA Sponges

LncRNAs are capable of acting as miRNA decoys, which diminish the suppressive effects of miRNAs on mRNAs and subsequently regulate glycolytic pathways within cancer cells [71]. For example, lncRNAs can modulate glycolysis genes in CRC by sponging miRNAs. The lncRNA LOXL1 has been found to induce HK2 expression and promote the resistance of CRC cells to apoptosis, invasion, migration and proliferation through the sequestration of miR‐1224‐5p/miR‐761 [72]. Similarly, Gao et al. reported that the inhibition of EGFR by the specific inhibitor erlotinib effectively enhanced the antitumor toxicity of 5‐FU by targeting the EGFR–lncRNA–FGD5‐AS1–miR‐330‐3p–HK2 pathway [73]. Moreover, the lncRNA MIR17HG functions as a ceRNA to regulate HK1 expression by sponging miR‐138‐5p, resulting in glycolysis in CRC cells and leading to invasion and liver metastasis [74]. The overexpression of lncARSR promoted the invasion, metastasis and glycolytic metabolic reprogramming of CRC cells through the miR‐34a‐5p/HK1 signalling pathway [75]. In addition to its influence on HK, the lncRNA–miRNA interaction also modulates PKM2 levels in CRC. Jin et al. pointed out that the silencing of TMPO‐AS1 promoted apoptosis in CRC cells while curbing their invasiveness, proliferation and migration through the regulation of the miR‐1270/PKM2 pathway [76]. High expression of XIST in CRC promotes proliferation, migration, invasion and resistance to 5‐FU and cisplatin by regulating miR‐137 and elevating the PKM2/PKM1 ratio [77]. β‐Enolase (ENO3) is a metalloenzyme that functions during glycolysis. LINC00174 was found to elevate ENO3 protein expression by binding to miR‐2467‐3p, promoting glycolysis, inflammation, proliferation, migration and invasion of colon cancer cells [78].

Moreover, the lncRNA–miRNA regulatory axis is involved in the control of glycolytic pathways in CRC, targeting pivotal oncogenes such as HIF‐1α. The level of lncRNA ANRIL was significantly increased in diabetic colon cancer specimens compared with nondiabetic colon cancer specimens. The lncRNA ANRIL can elevate glucose metabolism and CRC cell proliferation but inhibit cell apoptosis by sponging miR‐186‐5p, resulting in the subsequent upregulation of glycolytic enzymes, such as HIF‐1α^79^. Forkhead box protein M1 (FOXM1), a crucial transcriptional activator of LDHA and GLUT1, has potential effects on the glycolysis process in cancer [79]. Zhang and coworkers demonstrated that the suppression of the lncRNA MCF2L‐AS1 curtailed cellular glycolysis, migration, invasion and proliferation in CRC through its interaction with the miR‐874‐3p/FOXM1 signalling axis [80]. The forkhead transcription factor FOXC1 has been shown to modulate glycometabolism in CRC cells [81]. HIF1A‐AS2 can promote the proliferation, metastasis and aerobic glycolysis of CRC cells via the miR‐141‐3p/FOXC1 axis [82]. Additionally, AC105118.1 was shown to facilitate glycolysis and increase the resistance of CRC cells to oxaliplatin through the miR‐378a‐3p/KIF26B axis [83].

#### 4.2.2 LncRNAs Regulate Glycolysis by Interacting With Proteins

It has been shown that lncRNAs can exert their functions in a variety of ways, including via interactions with DNA, miRNAs and proteins [84]. LncRNA–protein interactions are involved in glycolysis in CRC. Recently, Yan and colleagues indicated that upregulated SLCC1 induced glycolysis activation and tumour growth in CRC by interacting with AHR and driving the transcriptional activation of HK2 [85]. LncRNA 495810 was found to interact directly with the PKM2 protein, stabilising it through modulation of the ubiquitin–proteasome degradation pathway [86, 87]. It has been reported that the lncRNA FEZF1‐AS1, which is upregulated in CRC patients, enhances CRC cell proliferation and metastasis via both glycolysis and STAT3 signalling by binding to PKM2 and increasing its stability [88]. Additionally, the lncRNA SNHG6 has been demonstrated to interact with RNPA1 and PKM, contributing to the PKM2/PKM1 shift and the growth of CRC [89]. Bian et al. reported that LINC01852, which is decreased in CRC, promotes the TRIM72‐mediated ubiquitination and degradation of SRSF5, curbing the SRSF5‐mediated alternative splicing of *PKM* and thereby downregulating the expression of PKM2 [90]. Hong et al. reported that the prevalence of *F. nucleatum* is related to elevated glucose metabolism in CRC patients and to increased expression of lncRNA ENO1‐IT1, which promotes CRC cell glucose metabolism and carcinogenesis by serving as a scaffold for KAT7 histone acetyltransferase to modulate ENO1 expression [91]. The lncRNA AC005392.2, which is induced by SOX2, promotes glycolysis and vasculogenic mimicry formation in CRC by increasing the SUMOylation‐mediated stability of the GLUT1 protein [92]. LncRNA SNHG15 knockdown inhibited CRC proliferation, resistance to 5‐FU chemotherapy and glycolysis, potentially through the regulation of TYMS, BCL2, GLUT1 and PKM2 expression [93]. These findings showed the interactions between lncRNAs and proteins, affecting glycolytic enzymes.

In addition to glycolysis genes, HIF‐1α and c‐MYC are pivotal factors that foster aerobic glycolysis and are regulated by the lncRNA–protein interactions. Tang et al. demonstrated that lncRNA GLCC1 overexpression supported cell survival, proliferation and glycolysis by directly binding with the chaperone HSP90 to prevent c‐Myc ubiquitination [94]. Depletion of the lncRNA LINRIS led to a reduction in the levels of IGF2BP2 (insulin‐like growth factor 2 mRNA‐binding protein 2), an emerging m6A ‘reader’, through the inhibition of K139 ubiquitination on IGF2BP2, which preserved its stability. Moreover, silencing of the lncRNA LINRIS attenuated glycolysis and cell proliferation via the IGF2BP2/c‐MYC axis [95]. Moreover, LINC01977 expression is significantly elevated in CRC tissues and enhances c‐Myc stability in an ERK‐mediated phosphorylation‐dependent manner, leading to enhancement in proliferation, metastasis and aerobic glycolysis of CRC cells [96]. Zuo et al. reported that the lncRNA MEG3, which is induced by vitamin D, could promote the ubiquitin‐caused degradation of c‐MYC, resulting in decreased glycolysis, proliferation and invasion of CRC cells [97]. A study by Li et al. revealed that lnc‐RP11‐536 K7.3 promotes CRC cell proliferation, glycolytic activity, angiogenic potential and chemoresistance. Mechanistic analysis revealed that lnc‐RP11‐536 K7.3 recruited SOX2 to transcriptionally activate the expression of USP7, leading to deubiquitylation and stabilisation of HIF‐1α [98].

In conclusion, the involvement of lncRNAs in modulating glycolysis, either directly or indirectly, offers a promising avenue for advancing our understanding of CRC metabolism and developing innovative treatment strategies.

### 4.3 CircRNA Dysregulation Is Related to Glycolysis in CRC

CircRNAs are a distinct category of endogenous ncRNAs, distinguished by their covalently closed circular structure, which distinguishes them from linear RNAs [99]. This circular configuration contributes to their stability and resistance to degradation, and they are abundant and conserved across various species [100]. A growing body of research has clarified the multiple functions of circRNAs within the context of neoplasms. They can act as miRNA sponges, binding and sequestering miRNAs to modulate gene expression and influence tumorigenesis [101]. Recent evidence has revealed the crucial roles of circRNAs in the glycometabolism of CRC (Table 3).

**TABLE 3:** Overview of circRNAs regulating glycolysis in CRC.

circRNAs	Expression	Regulatory mechanism	Phenotypes	Ref.
circ_0087862	up	ceRNA for miR‐512‐3p and increases HK2 expression	Proliferation, migration, invasion	[102]
Hsa_circ_0045932	up	ceRNA for miR‐873‐5p and increases HK2 expression	Proliferation, migration, invasion	[103]
circ‐CCS	up	ceRNA for miR‐874‐3p and increases HK2 expression	Oxaliplatin resistance, proliferation, migration, invasion	[104]
circ‐PITHD1	up	ceRNA for miR‐590‐5p and increases HK2 expression	Proliferation, invasion	[105]
circ_0053277	up	ceRNA for miR‐520 h and increases HK1 expression	growth angiogenesis metastasis	[126]
circVMP1	up	ceRNA for miR‐3167 and increases HKDC1 expression	Proliferation, metastasis	[106]
circAGFG1	up	ceRNA for miR‐7‐5p and increases PKM2 expression	Proliferation, oxaliplatin resistance	[107]
ciRS‐122	/	ceRNA for miR‐122 and increases PKM2 expression	oxaliplatin resistance	[108]
SPRY4‐IT1	up	interacts with PDK1 protein	Proliferation, migration, invasion	[109]
circ_0087862	up	ceRNA for miR‐296‐3p and increases PGK1 expression	Proliferation, migration, invasion	[110]
circHIF1A	/	ceRNA for miR‐361‐5p and increases HIF1α expression	Cetuximab resistance	[111]
circPLOD2	up	ceRNA for miR‐513a‐5p and increases SIX1‐mediated LDHA expression	Proliferation, migration, invasion	[112]

By sequestering miR‐512‐3p, the downregulation of circ_0087862 decreased cell viability, proliferation, migration/invasion and glycolysis while promoting cell apoptosis through HK2 [102]. Moreover, hsa_circ_0045932, circ‐CCS and circ‐PITHD1 were shown to function as sponges of miR‐873‐5p, miR‐874‐3p and miR‐590‐5p, respectively, increasing HK2 expression and enhancing CRC cell growth, invasion, dispersion and drug resisitance [103, 104, 105]. Moreover, circ_0053277 increased HK1 expression and HK1‐mediated glycolysis in CRC cells by negatively modulating the production of miR‐520 h^107^. Hexokinase domain component 1 (HKDC1) is a recently discovered enzyme and a potential fifth HK. The circRNA circVMP1 (hsa_circ_0006508), which is more abundant in CRC tissues, boosts the proliferation, spread and glycolytic metabolism of CRC cells by competitively binding to miR‐3167 and increasing HKDC1 levels [106]. Chen et al. reported increased circAGFG1 levels in oxaliplatin‐resistant CRC tissues and cells. They also demonstrated that the depletion of circAGFG1 decreased PKM2 expression to suppress glycolysis, oxaliplatin resistance and cellular growth in CRC through competitive binding with miR‐7‐5p [107]. Exosome‐mediated CiRS‐122 siRNA transport could curb glycolysis and reverse resistance to oxaliplatin by modulating the ciRS‐122/miR‐122/PKM2 pathway [108]. The lncRNA SPRY4‐IT1, which is highly upregulated in CRC tissues, fosters CRC cell growth and glycolysis by binding to the PDK1 protein, thereby increasing PDK1 protein levels [109]. A study by Xiao et al. revealed that circ_0087862 was highly expressed in CRC tissues and that the depletion of circ_0087862 curbed CRC cell growth, glycolysis and invasion, and increased apoptosis via the miR‐296‐3p/PGK1 axis [110]. Besides, circHIF1A and circPLOD2 sponge miR‐513a‐5p and miR‐361‐5p, respectively, and induce the overexpression of GLUT1 and LDHA [111, 112]. Overall, these results indicate that circRNAs participate in the regulation of glycolysis genes.

## 5 Conclusion and Prospects

Glycolysis is an important feature of various cancers including CRC, and increasing evidence indicates that multiple factors, such as ncRNAs, can modulate this pathway. This review of the role of miRNAs, lncRNAs and circRNAs in the regulation of glycolysis in CRC highlights the need for further investigations into the potential by which molecular mechanisms exist between ncRNAs and glycolytic pathways, which may help uncover new therapeutic strategies.

Previous reviews have reported that numerous ncRNAs are involved in the progression of CRC. An overall review by Yu et al. described the pivotal roles of exosomal ncRNAs in tumorigenesis and metastasis [113]. Luo et al. summarised the mechanisms by which ncRNAs regulate the initiation and development of chemoresistance to oxaliplatin in CRC [114]. Moreover, Zamer et al. discussed the genetic mutations and ncRNA‐based epigenetic modulations in the regulation of glycolysis and carcinogenesis in CRC [115]. However, few reports have systematically discussed the roles of ncRNAs in regulating glycolysis and the progression of CRC. Herein, we focused on how ncRNAs influence glycolysis through various molecular pathways and regulatory networks, such as by sequestering miRNAs, engaging with specific proteins and modulating key glycolytic enzymes or transcription factors to mediate metabolic reprogramming in CRC.

Notably, ncRNAs have specific expression patterns in various types of cancers, and their high specificity and accuracy make them potentially valuable biomarkers for cancer diagnosis. Mao et al. used glycolysis‐associated lncRNA profiles to develop a four‐lncRNA signature and a predictive nomogram for CRC outcomes [116]. Recently, miRNA–disease association (MDA) predictions have been implemented on the basis of the data fusion paradigm [117, 118]. The MDA model, which presents various relevant databases, experimental results, webservers and data fusion, contributes to disease prediction [119, 120]. Considering that glycolysis‐associated ncRNAs play key roles in the progression of CRC, developing a prediction model for the association between ncRNAs and CRC is of great clinical importance.

RNA binding proteins (RBPs) are a class of proteins involved in splicing, modification, transport, localisation, stability, degradation and translation of RNAs [121]. RBPs have been shown to be involved in CRC tumour progression and to be potential prognostic biomarkers for CRC patients [122, 123]. Notably, RBPs can interact with lncRNAs or circRNAs to regulate glycolysis. For example, the lncRNA HULC functions as an adaptor molecule that enhances the binding of LDHA and PKM2 to fibroblast growth factor receptor type 1 (FGFR1), leading to elevated phosphorylation of these two enzymes and consequently promoting glycolysis [124]. In the current review, we also noted that lncRNAs can interact directly with RBPs to modulate glycolysis in CRC. Hence, the expression levels of specific RBPs and their interacting RNAs may serve as potential diagnostic biomarkers for CRC. Moreover, targeting RBPs and their interactions with glycolytic enzymes or ncRNAs may be a promising therapeutic strategy for CRC.

One limitation of this review is that we did not discuss the roles of other types of ncRNAs, such as tRNAs, snRNAs and snoRNAs, in the regulation of glycolysis. Further investigations are needed. Moreover, the potential clinical significance of such glycolysis‐associated ncRNAs was not discussed. Further studies should explore how ncRNAs regulate glycolysis in different tumours to provide a new direction for future cancer treatment.

In summary, ncRNAs, as key glycolysis regulators, are likely to provide new and attractive targets for CRC therapy. Nevertheless, research into the molecular and clinical aspects of ncRNA‐mediated glycolysis regulation remains nascent. Additional research will emphasise the exploration of the specific functions and interrelations of different types of ncRNAs (such as miRNAs, lncRNAs and circRNAs) in CRC glycolysis. Moreover, clinical research on the modulation of glycolysis by ncRNAs is still in its infancy. Although some challenges remain, elucidating the roles and mechanisms of ncRNAs in modulating glycolysis may lead to novel therapeutic approaches in oncology.

## Author Contributions

**Liang Xu:** writing – original draft (equal), writing – review and editing (equal). **Yu Shen:** writing – original draft (equal), writing – review and editing (equal). **Chuanqiang Zhang:** funding acquisition (equal), methodology (equal). **Tongguo Shi:** supervision (equal), visualization (equal), writing – review and editing (equal). **Xuejuan Sheng:** supervision (equal), visualization (equal), writing – review and editing (equal).

## Conflicts of Interest

The authors declare no conflicts of interest.

## Data Availability

The datasets supporting the conclusions of this article are included within the article.
